# PD-L1-expressing neutrophils as a novel indicator to assess disease activity and severity of systemic lupus erythematosus

**DOI:** 10.1186/s13075-016-0942-0

**Published:** 2016-02-11

**Authors:** Qing Luo, Zikun Huang, Jianqing Ye, Yating Deng, Le Fang, Xue Li, Yang Guo, Hong Jiang, Beihua Ju, Qingshui Huang, Junming Li

**Affiliations:** Department of Clinical Laboratory, First Affiliated Hospital of Nanchang University, Nanchang, Jiangxi 330006 China; Department of medical college, Nanchang University, Nanchang, Jiangxi 330006 China; Department of Blood Transfusion, 521 Hospital of Ordnance Industry, Xi’an, Shanxi 710065 China

**Keywords:** Systemic lupus erythematosus, PD-L1, Neutrophils

## Abstract

**Background:**

It is well-known that increased frequency of neutrophils was found in patients with systemic lupus erythematosus (SLE). However, the immunomodulatory roles and mechanisms of neutrophils in SLE are poorly understood.

**Methods:**

Patients with SLE were recruited from the First Affiliated Hospital of Nanchang University. The medical history, clinical manifestations, physical examination, laboratory measurements, therapeutic regimen and treatment response were recorded. The expression of costimulatory molecules including programmed death 1 (PD-1), programmed death ligand 1 (PD-L1), T-cell immunoglobulin and mucin domain–containing protein 3 (Tim-3), CD40, T cell immunoreceptor with Ig and immunoreceptor tyrosine-based inhibitory domains (TIGIT), CD80 and CD86 on neutrophils were determined by flow cytometry. The frequencies of PD-L1-expressing neutrophils in patients with SLE were further analyzed for their correlation with markers of autoimmune response, inflammation, disease activity and severity of SLE.

**Results:**

The frequency of PD-L1-expressing neutrophils was significantly elevated in SLE patients compared to the healthy controls (P < 0.0001). The frequency of PD-L1-expressing neutrophils in patients with SLE was increased significantly in subjects with high ANA titre, high anti-nRNP/Sm, high levels of inflammatory markers and high SLE Disease Activity Index (SLEDAI) score. Furthermore, the percentages of PD-L1-expressing neutrophils were significantly decreased in SLE patients that received a 15-day regular treatment with corticosteroids and immunosuppressive drugs (P = 0.0075).

**Conclusion:**

The frequency of PD-L1-expressing neutrophils is elevates in patients with SLE, correlates with the disease activity and severity of SLE, and may serves as a negative feedback mechanism preventing potential tissue damage caused by excessive autoimmune responses in patients with SLE.

## Article headings

PD-L1 expression on neutrophils in SLE

## Background

Systemic lupus erythematosus (SLE) is a systemic autoimmune syndrome with unclear etiology that affects multiple organs and afflicts mostly women of childbearing age. The skin, blood vessels, kidneys, central nervous system, and joints are common targets of inflammation at onset or during the course of the disease. The development of SLE is attributed to disruptions in adaptive immunity, triggered by genetic predisposing factors and environmental triggers, which lead to the loss of tolerance to self-antigens. Indeed, B and T lymphocytes play prominent pathogenic roles in the development and progression of SLE [1, 2].

However, over the last few decades, evidence has clearly indicated the disorder of innate immune responses in the development of SLE [3–6]. Neutrophils, the most abundant white blood cells in humans, play crucial roles as sentinels and first-line of defense against pathogens in the innate immune system. Recent evidence indicates that neutrophils may play an important role in the induction of autoimmune responses and organ damage in SLE [7, 8]. Lupus neutrophils have been reported to have altered functional properties, including diminished phagocytic capabilities, aberrant clearance of apoptotic material, decreased responsiveness to cytokines, increased aggregation, and intravascular activation [9]. Nevertheless, the role of lupus neutrophils in SLE pathogenesis has not been well-elucidated.

Programmed death ligand 1 (PD-L1) is one of the costimulatory molecules in the B7 family, which functions as an immunomodulatory molecule. The engagement of PD-L1 with its receptor, programmed death 1 (PD-1), delivers inhibitory signals to target cells such as activated T cells and B cells, and thus helps to maintain the balance between effective immunity, tolerance and immunopathology [10]. PD-L1 is broadly expressed on a variety of immune cells, including T cells, B cells, dendritic cells, and monocytes. Recent evidence indicates that PD-L1 is also expressed on neutrophils and is associated with the development of numerous diseases, including human immunodeficiency virus [11], sepsis [12], Burkholderia pseudomallei-infected disease [13], and tuberculosis [14]. However, the frequency and roles of PD-L1-expressing neutrophils in SLE has not been established. In the present study, we determined the frequency of PD-L1-expressing neutrophils in patients with SLE and tested the hypothesis that their frequency correlates with the activity and severity of SLE.

## Methods

### Subjects

Patients (n = 77) who patients fulfilled the revised American College of Rheumatology criteria for SLE [15] were enrolled from the First Affiliated Hospital of Nanchang University from June 2014 to July 2015. Disease activity was assessed by the SLE disease activity index (SLEDAI) [16]. In addition, this study included 43 healthy controls without inflammatory or autoimmune diseases, who were unrelated to the patients. The study was approved by the Ethics Committee of the First Affiliated Hospital of Nanchang University and was carried out in compliance with the Helsinki Declaration. Informed consent was obtained from all the participants before they entered the study.

### Flow cytometry analysis

Peripheral blood was drawn and analyzed immediately for the molecular phenotypes of neutrophils, using flow cytometry. The following antibodies were used: phycoerythrin-Texas Red (ECD)-conjugated anti-CD3, phycoerythrin-Cyanin 5 (PC5)-conjugated anti-CD15 (BD Biosciences, San Diego, CA, USA), phycoerythrin (PE)-conjugated anti-PD1, anti-T-cell immunoglobulin and mucin domain-containing protein 3 (Tim3), anti-T cell immunoreceptor with Ig and immunoreceptor tyrosine-based inhibitory domains (TIGIT), anti-CD86, and anti-PDL1, fluorescein isothiocyanate (FITC)-conjugated anti-CD80 (MIH clones, e Bioscience, San Diego, CA, USA). The neutrophils were identified as CD15^+^CD3^−^ populations [11] and the membranous markers were detected by flow cytometry with triple staining. Briefly, 50 μL of fresh heparinized whole blood were incubated simultaneously with 5 μL ECD-conjugated anti-CD3, 5 μL PC5-conjugated anti-CD15 and 5 μL fluorescence-conjugated antibodies targeting other membranous molecules on ice in the dark for 30 minutes. Cells incubated with PE- and FITC- conjugated mouse IgG were used as isotype controls. All flow samples were analyzed with a CYTOMICS FC 500 flow cytometer (Beckman Coulter Inc., Brea, CA, USA) and associated software programs (CXP).

### Autoantibodies measurement

The antinuclear antibodies (ANA) were detected using the indirect immunofluorescence method with a commercially available diagnostic kit (EUROIMMUN, Germany) according to the manufacturer’s instructions [17]. Serum samples from SLE patients were prepared at various dilution factors as follows: 1:100, 1:320, 1:640 and 1:1000. The sample was defined as ANA-positive when the signal could be detected with the serum diluted at 1:100. Anti-double-stranded DNA (Anti-dsDNA) Abs of IgG in serum were measured by commercially available ELISA kits (Kexin, Shanghai, China). Anti-extractable nuclear antigens (ENAs) antibodies including anti-Sjogren’s syndrome-related antigen A (anti-SSA), anti-Sjogren’s syndrome-related antigen A (anti-SSB), anti-Ro52, anti-Smith (anti-Sm), anti-nRNP/Sm, anti-ribosomal ribonucleoprotein (anti-rRNP), and anti-nucleosome antibody were determined by immunoenzyme dot assay (EUROIMMUN, Germany) according to the manufacturer’s instructions. The results of anti-ENA detection were determined as negative (–) or positive (+, ++, +++) by EUROBlotOne.

### Serum IgG, C-reactive protein (CRP), Complement 3 (C3) and C4 measurement

The concentrations of serum immunoglobulin G (IgG), CRP, C3, and C4 were determined by nephelometry methods according to the instructions described by the manufacturer (IMMUNE800, Beckman Coulter).

### Erythrocyte sedimentation rate (ESR) and routine urine and blood measurements

ESR and routine urine and blood measurements were determined according to the instructions described by the manufacturer.

### Statistical analysis

Statistical analysis and graphic presentation were carried out with GraphPad Prism version 5.0 (GraphPad Software, San Diego, CA, USA). The *t* test was used when normal data distribution was confirmed; otherwise, the nonparametric Mann–Whitney test was used to analyze the data. The paired *t* test was performed for evaluation of changes with treatment in the group of nine patients. Likewise, the Pearson method or the nonparametric Spearman method was used for correlation analysis. A value of *P* <0.05 was considered statistically significant.

## Results

### Characteristics of study subjects

Characteristics of SLE patients and healthy controls enrolled in this study are included in Table 1. There were no significant differences between patients and healthy controls in age or gender. Patients with SLE were classified into a group with inactive disease (SLEDAI score 0–5) and a group with active disease (SLEDAI score ≥6) [16]. Overall, 20 % of SLE patients have active disease. All patients received corticosteroids and immunosuppressive drugs. Among them, 10 patients were monitored before and after receiving regular treatment.

**Table 1:** Baseline characteristics of systemic lupus erythematosus patients

Categories	SLE patients (n = 77)	Healthy controls (n = 43)
General conditions		
Female,%	91 %	77 %
Age, years, average ± SD	35 ± 13	32 ± 11
SLEDAI, median (range)	4 (0–14)	
Clinical features, number of patients		
Fever	5	
Cutaneous manifestations	12	
Oral ulcer	3	
Alopecia	5	
Arthritis	11	
Raynaud’s phenomenon	5	
Effusion	3	
Renal involvement	27	
Laboratory parameters, number of patients		
Leucopenia	8	
Erythrocytopenia	20	
Thrombocytopenia	5	
Anemia	29	
Pyuria	13	
Hematuria	6	
Proteinuria	16	
Anti-dsDNA(+)	27	
Increased ANA (>1:320)	27	
Anti-ENA (39 patients)		
Anti-Sm	15	
Anti-Ro52	24	
Anti-nRNP/Sm	24	
Anti-Rrnp	16	
Anti-nucleosome	13	
Anti-SSA	29	
Anti-SSB	10	
Decreased C3/C4	38/31	
Increased IgG	24	
Elevated ESR/CRP	38/10	

*SLE* systemic lupus erythematosus, *SLEDAI* SLE disease activity index, *Anti-dsDNA* anti double-stranded DNA, *ANA* antinuclear antibodies, *C3* complement 3, *C4* complement 4, *ESR* erythrocyte sedimentation rate, *CRP* C-reactive protein, *Anti-SSA* anti-Sjogren’s syndrome-related antigen A, *Anti-SSB* anti-Sjogren’s syndrome-related antigen B, *ENA* anti-extractable nuclear antigen, *Sm* Smith, *RNP* ribonucleoprotein, *rRNP* ribosomal RNP

### Elevated frequency of PD-L1-expressing neutrophils in patients with SLE

**Fig. 1:**
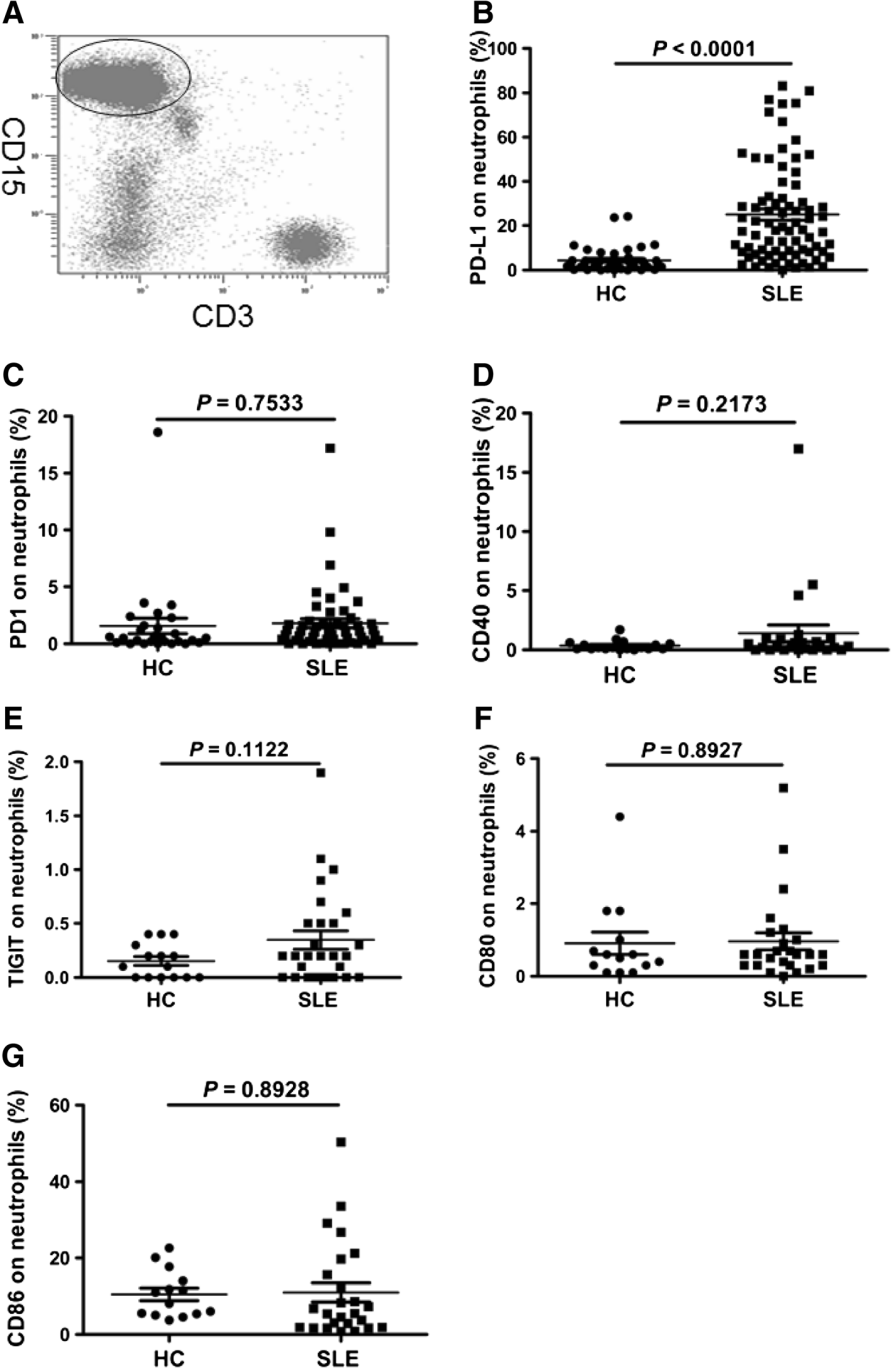
Expression of programmed death ligand 1 (*PD-L1*), programmed death 1 (*PD*1), CD40, T cell immunoreceptor with Ig and immunoreceptor tyrosine-based inhibitory domains (*TIGIT*), CD80 and CD86 in neutrophils. **a** Gates were set on neutrophils based on CD15 and CD3; neutrophils were defined as CD15 ^+^CD3^−^ forward scatter (FSC) high, side scatter (SSC) high. **b** Systemic lupus erythematosus (*SLE*) patients had elevated frequency of PD-L1-expressing neutrophils compared with healthy controls (*HC*) (*P* <0.0001). **c** The frequency of PD1-expressing neutrophils was similar in HC and SLE patients (*P* = 0.7533). **d** The frequency of CD40-expressing neutrophils was similar in HC and SLE patients (*P* = 0.2173). **e** The frequency of TIGIT-expressing neutrophils was similar in HC and SLE patients (*P* = 0.1122). **f** The frequency of CD80-expressing neutrophils was similar in HC and SLE patients (*P* = 0.8927). **g** The frequency of CD86-expressing neutrophils was similar in HC and SLE patients (*P* = 0.8928)

The neutrophils were identified in peripheral blood as CD15^+^ CD3^−^ populations and analyzed by flow cytometry for the expression of costimulatory molecules including PD-1, PD-L1, Tim-3, TIGIT, CD40, CD80 and CD86. Data showed that the frequency of PD-L1-positive neutrophils was significantly elevated in patients with SLE compared to healthy volunteers (Fig. 1, *P* <0.0001). No significant difference was observed in the frequency of PD1-, CD40-, TIGIT-, CD80-, or CD86-expressing neutrophils between SLE individuals and healthy controls (Fig. 1). There was no apparent expression of Tim-3 by neutrophils.

### The frequency of PD-L1-expressing neutrophils correlated with markers of autoimmune response

**Fig. 2:**
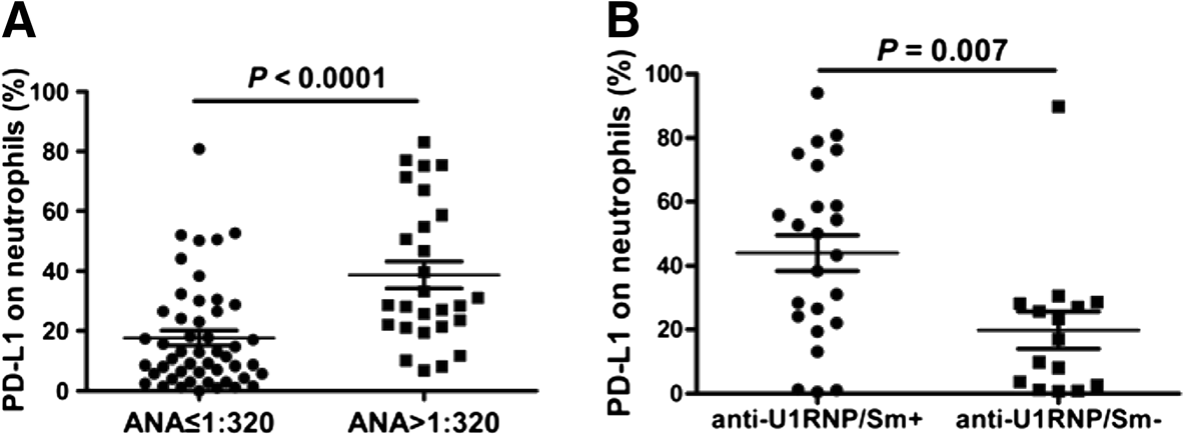
Correlation between the frequency of programmed death ligand 1 (*PD-L1*)-expressing neutrophils with autoantibody. **a** The frequency of PD-L1-expressing neutrophils was significantly increased in systemic lupus erythematosus (SLE) patients with a high antinuclear antibodies (*ANA*) titer (ANA >1:320) compared to SLE patients with low-to-medium ANA titer (ANA ≤1:320) (*P* <0.0001). **b** The frequency of PD-L1-expressing neutrophils was significantly increased in SLE patients who were positive to anti ribonucleoprotein/Smith (*anti-nRNP/Sm*) (*P* = 0.007)

The hallmark antibodies of SLE, including ANA, anti-dsDNA and anti-ENAs, were determined and analyzed for their relationship with the frequency of PD-L1-expressing neutrophils in this study. Data showed that all patients were positive for ANA and 27 patients were positive for anti-dsDNA: 39 patients were tested for anti-ENAs among all recruited SLE patients and 38 patients were positive for at least one anti-ENA. Patients with SLE were then divided into a low-to-medium titer group (titer ≤1:320) and a high titer group (titre >1:320) according to the results of ANA determination. As shown in Fig. 2a, the frequency of PD-L1-expressing neutrophils was significantly increased in patients with high ANA titer compared to patients with low-to-medium ANA titer. There was a trend towards elevated frequency of PD-L1-expressing neutrophils in patients who were positive to anti-dsDNA, but the difference was not significant (data not shown). Moreover, we investigated correlation between the frequency of PD-L1-expressing neutrophils and anti-ENA detection, including anti-SSA, anti-SSB, anti-Ro52, anti-Sm, anti-nRNP/Sm, anti-rRNP, and anti-nucleosome. As shown in Fig. 2b, the frequency of PD-L1-expressing neutrophils was significantly increased in patients with positive anti-nRNP/Sm compared to patients with negative anti-nRNP/Sm. No obvious correlation was observed between the frequency of PD-L1-expressing neutrophils and other anti-ENAs (data not shown). These results showed that the elevated frequency of PD-L1-expressing neutrophils is correlated with the markers of autoimmune response, suggesting that PD-L1-expressing neutrophils may be associated with the pathogenesis of SLE.

### The frequency of PD-L1-expressing neutrophils correlated with markers of inflammation

**Fig. 3:**
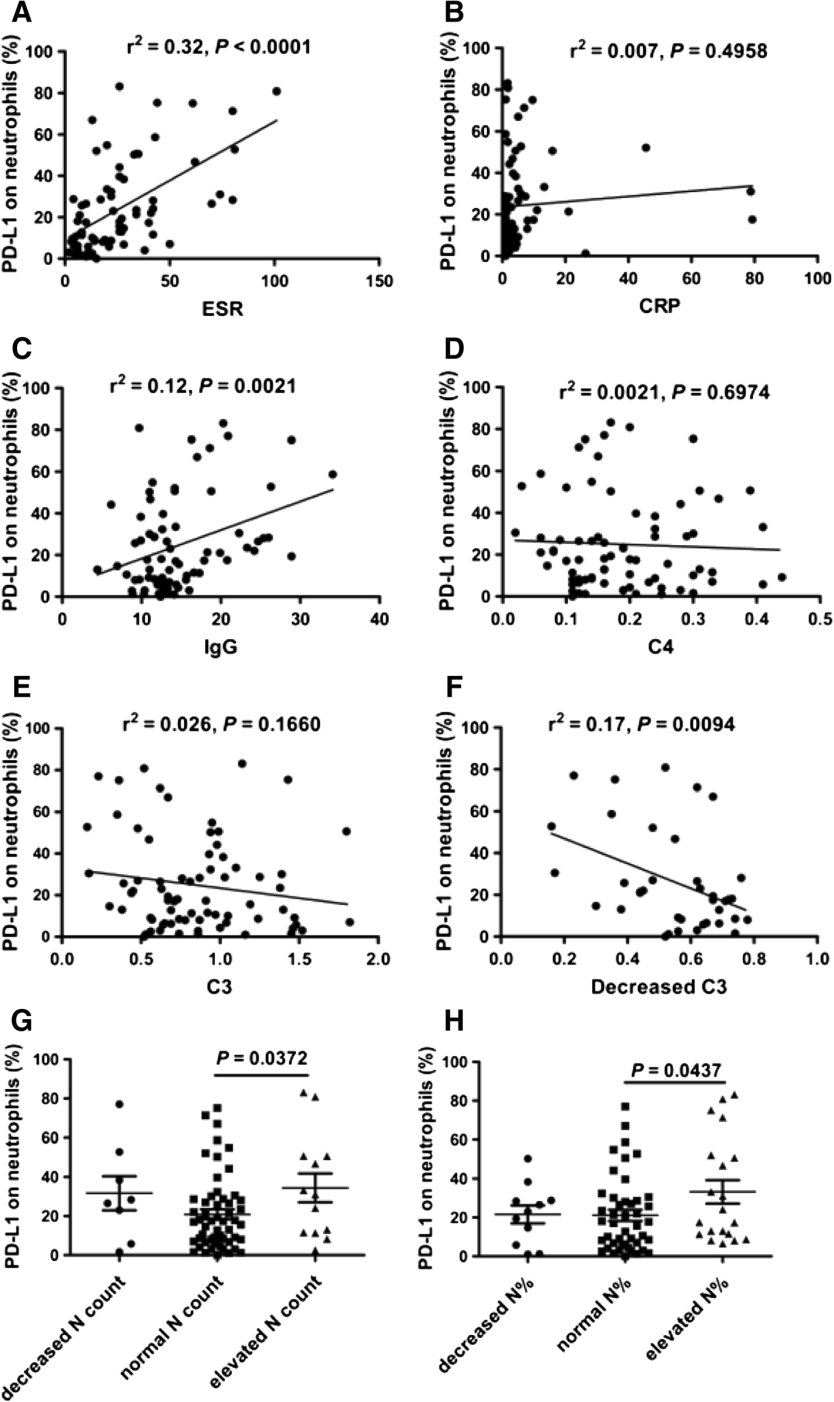
Correlation of frequency of programmed death ligand 1 (*PD-L1*)-expressing neutrophils with inflammatory markers. **a** The frequency of PD-L1-expressing neutrophils in systemic lupus erythematosus (SLE) patients correlated significantly with erythrocyte sedimentation rate (*ESR*) (*r* ^2^ = 0.32, *P* <0.0001). **b** The frequency of PD-L1-expressing neutrophils in SLE patients was not correlated with C-reactive protein (*CRP*) (*r* ^2^ = 0.007, *P* = 0.4958). **c** The frequency of PD-L1-expressing neutrophils in SLE patients correlated significantly with immunoglobulin G (*IgG*) (*r* ^2^ = 0.12, *P* <0.0021). **d** The frequency of PD-L1-expressing neutrophils in SLE patients was not correlated with Complement 4 (*C4*) (*r* ^2^ = 0.0021, *P* = 0.6974). **e** The frequency of PD-L1-expressing neutrophils in SLE patients was not correlated with C3 (*r* ^2^ = 0.026, *P* = 0.1660). **f** The frequency of PD-L1-expressing neutrophils in SLE patients was correlated negatively with decreased C3 (*r* ^2^ = 0.17, *P* = 0.0094). **g** The frequency of PD-L1-expressing neutrophils was significantly increased in SLE patients with elevated neutrophil (*N*) count compared to those with normal N count (*P* = 0.0372). **h** The frequency of PD-L1-expressing neutrophils was significantly increased in SLE patients with elevated neutrophil percentages (*N%*) compared to those with normal N% (*P* = 0.0437)

Patients with SLE are frequently accompanied by the elevated levels of inflammatory markers. In order to investigate the correlation between the frequency of PD-L1-expressing neutrophils and inflammatory markers, the markers of inflammation, including ESR, neutrophil count and neutrophil percentage, serous CRP, immunoglobulin and Complement were determined and analyzed for their correlation with the frequency of PD-L1-expressing neutrophils in patients with SLE. As shown in Fig. 3, there was positive correlation between the frequency of PD-L1-expressing neutrophils, and ESR and IgG. No obvious correlation was observed between the frequency of PD-L1-expressing neutrophils and other inflammatory markers. However, the frequency of PD-L1-expressing neutrophils was significantly elevated in SLE patients with elevated neutrophil counts (Fig. 3g) and neutrophil percentages (Fig. 3h). Furthermore, we made the surprising observation that the frequency of PD-L1-expressing neutrophils was inversely associated with decreased C3 (Fig. 3f). These results indicate that the frequency of PD-L1-expressing neutrophils is associated with markers of inflammation.

### The frequency of PD-L1-expressing neutrophils correlated with disease activity and severity of SLE

**Fig. 4:**
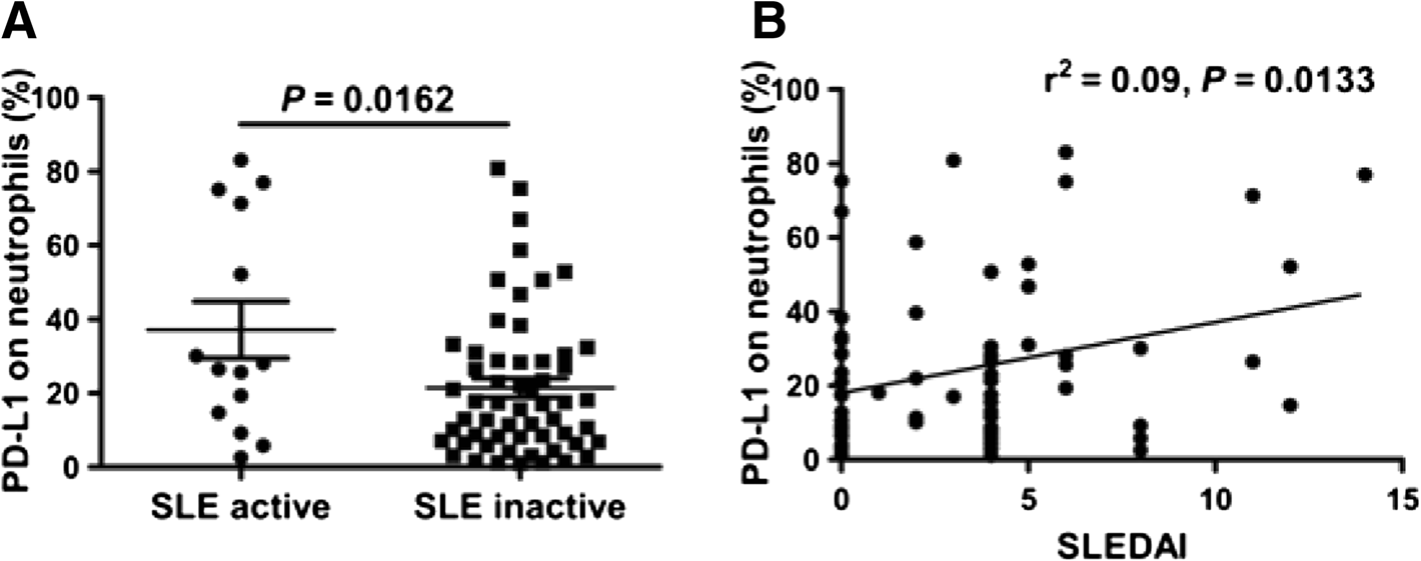
Correlation of frequency of programmed death ligand 1 (PD-L1)-expressing neutrophils with disease activity. **a** The frequency of PD-L1-expressing neutrophils in systemic lupus erythematosus (*SLE*) patients was significantly increased in patients with active SLE compared to those with inactive SLE (*P* = 0.0162). **b** The frequency of PD-L1-expressing neutrophils in SLE patients correlated significantly with systemic lupus erythematosus disease activity index (*SLEDAI*) (*r* ^2^ = 0.09, *P* = 0.0133)

The aforementioned results demonstrate that the frequency of PD-L1-expressing neutrophils was correlated with markers of autoimmune response and inflammation. Some of these markers, such as anti-dsDNA, IgG and C3 are traditionally valuable for monitoring disease activity in patients with SLE [18–20]. Thus, patients with SLE were further classified as patients with active or inactive disease according to the SLEDAI and analyzed for the relationship with the frequency of PD-L1-expressing neutrophils. Data showed that the frequency of PD-L1-expressing neutrophils in patients with active SLE was significantly higher compared with patients with inactive SLE (*P* = 0.0162) (Fig. 4a). Furthermore, we found that there was a positive correlation between the frequency of PD-L1-expressing neutrophils and the SLEDAI score (*r*^2^ = 0.09, *P* = 0.0133) (Fig. 4b), which demonstrated that the frequency of PD-L1-expressing neutrophils is correlated with disease activity in SLE.

**Fig. 5:**
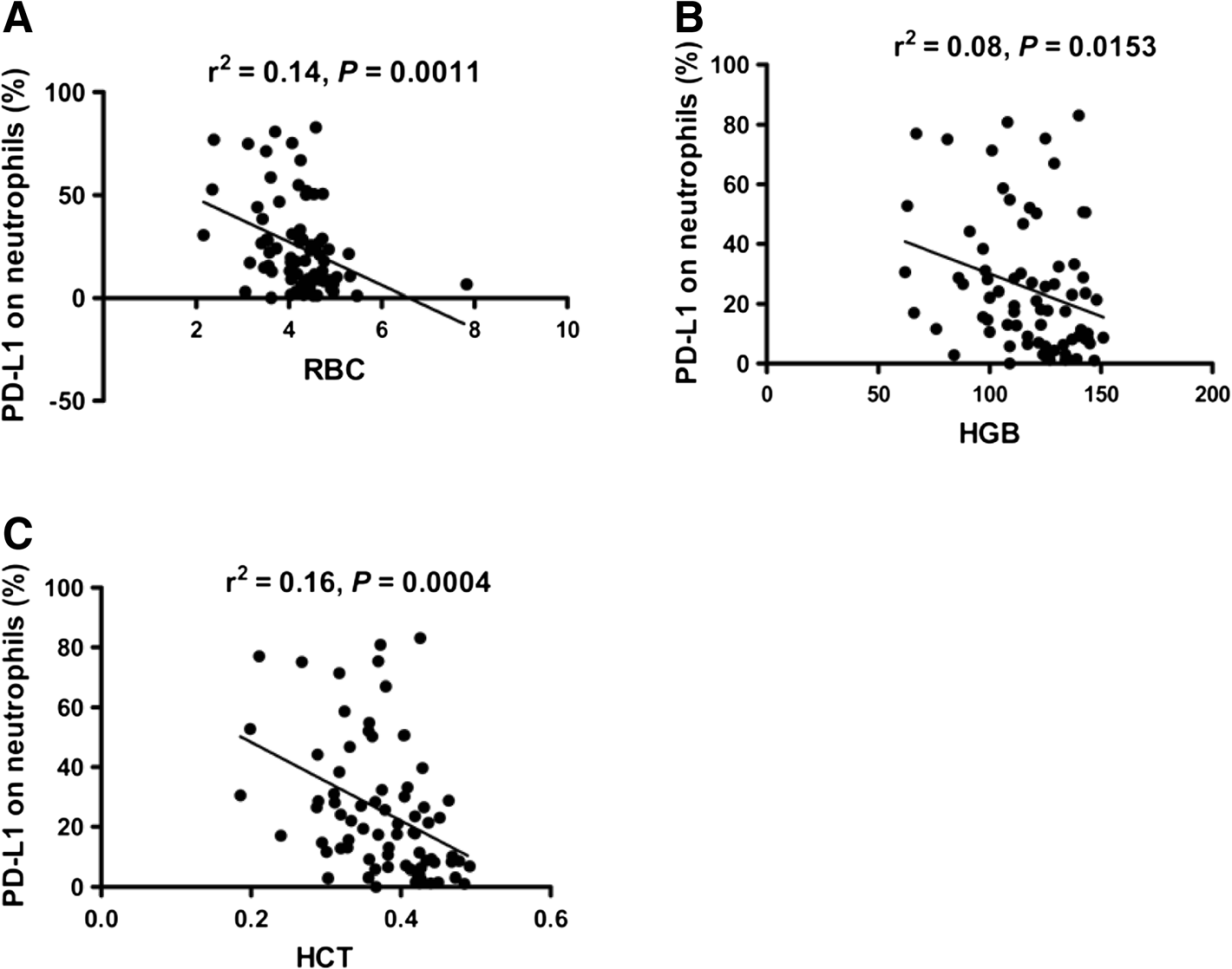
Association between the frequency of programmed death ligand 1 (*PD-L1*)-expressing neutrophils with red blood cells (*RBC*), hemoglobin (*HGB*) and hematocrit (*HCT*). **a** The frequency of PD-L1-expressing neutrophils in systemic lupus erythematosus (SLE) patients correlated negatively with RBC (*r* ^2^ = 0.14, *P* = 0.0011). **b** The frequency of PD-L1-expressing neutrophils in SLE patients correlated negatively with HGB (*r* ^2^ = 0.08, *P* = 0.0153). **c** The frequency of PD-L1-expressing neutrophils in SLE patients correlated negatively with HCT (*r* ^2^ = 0.16, *P* = 0.0004)

Next, the clinical features of patients with SLE, including fever, cutaneous manifestations, oral ulcer, alopecia, arthritis, Raynaud’s phenomenon, effusion, renal involvement and hematologic disorder were analyzed and correlation tested between these and the frequency of PD-L1-expressing neutrophils. Results showed that the frequency of PD-L1-expressing neutrophils was significantly elevated in SLE patients with fever, cutaneous manifestations and hematuria, but not in renal involvement (characterized by proteinuria, hematuria, or >5 leukocytes/high power field (hpf) excluding infection [21–23]), proteinuria and pyuria (Table 2). PD-L1-expressing neutrophils were also more frequently detected in patients with leucopenia or thrombocytopenia (Table 2). Additionally, we made the observation that the frequency of PD-L1-expressing neutrophils was inversely associated with red blood cell (RBC) count (*r*^2^ = 0.14, *P* = 0.0011) (Fig. 5a), hemoglobin (HGB) level (*r*^2^ = 0.08, *P* = 0.0153) (Fig. 5b) and hematocrit (HCT) (*r*^2^ = 0.16, *P* = 0.0004) (Fig. 5c), indicated that the frequency of PD-L1-expressing neutrophils was correlated with erythrocytopenia and anemia.

**Table 2:** Association between frequency of programmed death ligand 1 (PD-L1)-expressing neutrophils and clinical features

Clinical features (number)		Case	PD-L1-expressing neutrophils (%)	*P*
Cutaneous manifestations (71)	Yes	12	44.18 ± 8.156	0.0046
	No	59	20.55 ± 2.419	
Arthritis (71)	Yes	11	35.29 ± 6.522	0.0805
	No	60	22.59 ± 2.822	
Alopecia (71)	Yes	4	30.93 ± 7.235	0.5580
	No	67	24.18 ± 2.755	
Fever (71)	Yes	5	47.60 ± 9.637	0.0148
	No	66	22.82 ± 2.629	
Oral ulcer (71)	Yes	3	41.80 ± 9.211	0.1702
	No	68	23.80 ± 2.688	
Raynaud's phenomenon (71)	Yes	5	42.90 ± 10.20	0.0543
	No	66	23.17 ± 2.665	
Serositis (71)	Yes	3	25.37 ± 2.978	0.9492
	No	68	24.53 ± 2.744	
Renal involvement (71)	Yes	27	21.71 ± 4.256	0.3998
	No	44	26.31 ± 3.355	
Pyuria (71)	Yes	13	15.28 ± 5.781	0.0948
	No	58	26.64 ± 2.899	
Hematuria (71)	Yes	6	43.33 ± 12.92	0.029
	No	65	22.83 ± 2.550	
Proteinuria (71)	Yes	16	22.41 ± 5.084	0.6627
	No	55	25.19 ± 3.074	
Erythrocytopenia (71)*	Yes	20	36.06 ± 5.64	0.0161
	No	51	21.90 ± 2.837	
Leucopenia (59)*	Yes	8	40.30 ± 10.01	0.0232
	No	51	21.75 ± 2.751	
Thrombocytopenia (68)*	Yes	5	42.34 ± 11.31	0.0337
	No	63	21.84 ± 2.517	
Anemia (75)	Yes	19	37.47 ± 5.711	0.0041
	No	56	20.82 ± 2.648	

The *t* test was used when the data distribution was confirmed as normal, otherwise, the nonparametric Mann–Whitney test was used to analyze the data. *P* <0.05 was considered as statistically significant. *SLE patients with increased cell counts of RBC, WBC and PLT were excluded

**Fig. 6:**
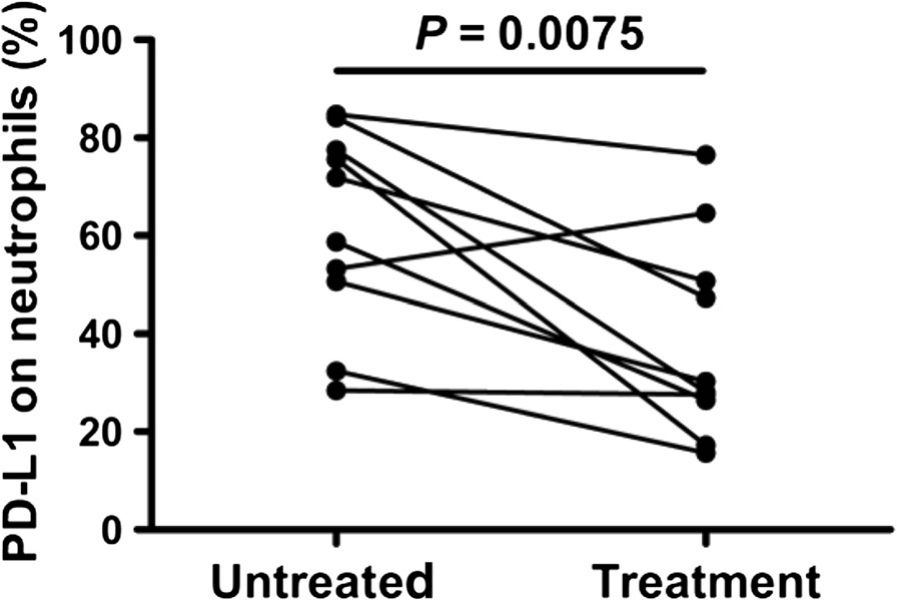
Influence of regular treatment on the frequency of programmed death ligand 1 (*PD-L1*)-expressing neutrophils. Decreased frequency of PD-L1-expressing neutrophils is shown in systemic lupus erythematosus patients following treatment with corticosteroids and immunosuppressive drugs (*P* = 0.0075)

Subsequently, we performed a 15-day follow-up evaluation in 10 SLE patients who received regular treatment with corticosteroids and immunosuppressive drugs. The clinical response and frequency of PD-L1-expressing neutrophils were monitored during the course of treatment. Notably, the frequency of PD-L1-expressing neutrophils in nine of the SLE patients decreased following treatment when compared with those prior to treatment, and one SLE patient had increased frequency of PD-L1-expressing neutrophils. As shown in Fig. 6, after treatment there was a significant difference (*P* = 0.0075). These results show that the frequency of PD-L1 expression on neutrophils correlates with disease severity.

## Discussion

Neutrophils represent the most abundant leukocyte population and traditionally recognized as one of essential effector cells of the innate immune system in humans. In recent years, an increasing interest in the role of neutrophils in interacting with and regulating the adaptive immune response has emerged [11–14, 24, 25]. It is well-known that various SLE biomarkers are neutrophil-related and increased frequencies of neutrophils are found in SLE. However, the immunomodulatory roles and mechanisms of neutrophils in the onset and development of SLE are poorly understood. It is well-known that the expression of costimulatory molecules plays an important role in determining the activation status and function of immune cells. Some costimulatory molecules, especially some immunosuppressive costimulatory molecules, such as PD1, PD-L1, Tim-3 and TIGIT, have been reported to have abnormal expression on peripheral T cells, B cells, monocyte or natural killer cells in patients with SLE [26–30]. In this study, for the first time, we investigated the expression of CD80, CD86, PD1, PD-L1, Tim-3, CD40 and TIGIT on neutrophils from patients with SLE, and showed that the frequency of PD-L1-expressing neutrophils was significantly increased in patients with SLE compared with healthy individuals. Moreover, our research revealed that the frequency of PD-L1-expressing neutrophils was associated with disease activity and severity of SLE.

SLE is one of systemic autoimmune diseases characterized by elevated autoimmune antibodies. In this study, the serous levels of ANA, anti-dsDNA and anti-ENA, including anti-SSA, anti-SSB, anti-Ro52, anti-Sm, anti-nRNP/Sm, anti-rRNP, and anti-nucleosome, the hallmark antibodies of SLE, were first determined and analyzed for their relationship with the frequency of PD-L1-expressing neutrophils. Data showed that the frequency of PD-L1-expressing neutrophils was significantly increased in patients with a high ANA titer and positive anti-nRNP/Sm, suggested that PD-L1-expressing neutrophils may be associated with autoimmune responses in SLE. However, although there was a tendency towards an increase in patients with positive anti-dsDNA, the frequency of PD-L1-expressing neutrophils was not significantly correlated with the anti-dsDNA titer. As the ds-DNA titer would decrease following therapy with corticosteroids and immunosuppressive drugs, the poor correlation between the frequency of PD-L1-expressing neutrophils and anti-dsDNA may be due to the fact that 87 % of SLE patients had received therapy prior to participation in the study. It is well-known that autoimmune response is a kind of chronic inflammation against self-antigens. So the correlation between the frequency of PD-L1-expressing neutrophils and inflammatory markers was analyzed. Our results showed that the frequency of PD-L1-expressing neutrophils was positively related to ESR and IgG, but inversely associated with decreased C3.

Patients with SLE have a complex array of abnormalities involving their immune system. Hematologic disorders, such as leukopenia, erythrocytopenia, anemia or thrombocytopenia, are one of the most frequent types of disorder in patients with SLE [31–33] and are associated with disease activity in SLE. In this study, we observed that PD-L1 expression on neutrophils was inversely associated with anemia and erythrocytopenia, as marked by reduced HGB level, HCT and RBC count, respectively. In addition, results showed that the frequency of PD-L1-expressing neutrophils was significantly elevated in SLE patients with fever and cutaneous manifestations. Although, there was no correlation between the frequency of PD-L1-expressing neutrophils and renal involvement, the results showed that the frequency of PD-L1-expressing neutrophils was significantly elevated in SLE patients with hematuria, but not with proteinuria or pyuria. It may be due to the relatively poor sensitivity of the method used for urinary protein detection. The dry chemical method was used for proteinuria and hematuria analysis in this study. The sensitivity is 0.1 g albumin/L and 0.3 mg hemoglobin/L for proteinuria and hematuria analysis, respectively. These results suggest that the frequency of PD-L1-expressing neutrophils may be correlated with disease activity in SLE. Subsequent results from the SLEDAI classification of SLE patients confirmed our speculations. Thus, we established the correlation between the frequency of PD-L1-expressing neutrophils and disease activity in SLE.

To further investigate the clinical significance of the PD-L1-expressing neutrophils in SLE, we performed a 15-day follow-up evaluation in 10 SLE patients who received regular treatment with corticosteroids and immunosuppressive drugs. Data showed that the percentages of PD-L1-expressing neutrophils decreased in nine of these patients and were increased in one patient after 15-day treatment with corticosteroids and immunosuppressive drugs. Due to the complex characteristics of SLE, both the clinical features and response to treatment are diverse and variable in patients with SLE [34]. Further analysis showed that the patient with increased percentages of PD-L1-expressing neutrophils had no improvement either in the SLEDAI score or clinical manifestation. Thus, these results demonstrated that the frequency of PD-L1-expressing neutrophils is correlated with disease severity in SLE.

Previously, an immunosuppressive population of neutrophils has been identified in peripheral blood of humans in particular events, such as the presence of chronic inflammation and tumors [35, 36]. This population of neutrophils displays a remarkable ability to suppress T cell-mediated immune response by multiple mechanisms, including production of reactive oxygen species (ROS) and arginase-1 [11, 37], and is speculated to serve as a negative feedback mechanism preventing potential tissue damage caused by excessive immune response. Considering the immunosuppressive feature of PD-L1 and the fact that the frequency of PD-L1-expressing neutrophils is associated with disease activity and severity in SLE, our research suggests that the increased frequency of PD-L1-expressing neutrophils may serve as a negative feedback mechanism, preventing potential tissue damage caused by excessive autoimmune responses in patients with SLE.

## Conclusions

To our knowledge, this is the first report on the characteristics of PD-L1-expressing neutrophils in SLE. Additionally, our research established correlation between the frequency of PD-L1-expressing neutrophils and disease activity and severity in SLE, which might improve our understanding of the role of neutrophils in SLE.

## Abbreviations

Abs: antibodies
ANA: antinuclear antibodies
Anti-dsDNA: anti-double-stranded DNA
Anti-SSA: anti-Sjogren’s syndrome-related antigen A
Anti-SSB: anti-Sjogren’s syndrome-related antigen B
C3: Complement 3
C4: Complement 4
CRP: C-reactive protein
ELISA: enzyme-linked immunosorbent assay
ENA: anti-extractable nuclear antigen
ESR: erythrocyte sedimentation rate
HCT: hematocrit
HGB: hemoglobin
IgG: immunoglobulin G
PD-1: programmed death 1
PD-L1: programmed death ligand 1
RBC: red blood cell
RNP: ribonucleoprotein
rRNP: ribosomal RNP
SLE: systemic lupus erythematosus
SLEDAI: SLE disease activity index
Sm: Smith
TIGIT: T cell immunoreceptor with Ig and immunoreceptor tyrosine-based inhibitory domains
Tim-3: T-cell immunoglobulin and mucin domain-containing protein 3
